# Genomic characterization of pulmonary sarcomatoid adenocarcinoma: a paired whole-exome sequencing study of carcinomatous and sarcomatous components

**DOI:** 10.3389/fonc.2026.1796428

**Published:** 2026-04-22

**Authors:** Jing Lin, Yuzhong Yang, Mengqing Liu, Fan Yang, Shaofeng Jiang, Longkuan Xu, Xiang Zheng, Hui Wei, Xuyan Wen, Guining Xu, Ruolan Weng, Jinhua Zheng, Shengjun Xiao

**Affiliations:** 1 Guangxi Key Laboratory of Metabolic Reprogramming and Intelligent Medical Engineering for Chronic Diseases, The Second Affiliated Hospital of Guilin Medical University, Guilin, China; 2 Department of Pathology, The First Affiliated Hospital of Guilin Medical University, Guilin, China; 3 College of Humanities and Management, Guilin Medical University, Guilin, China; 4 Guangxi Key Laboratory of Tumor Immunology and Microenvironmental Regulation, Guilin Medical University, Guilin, China; 5 Department of Pathology, The Second Affiliated Hospital of Guilin Medical University, Guilin, China

**Keywords:** epithelial-mesenchymal transition, intratumor heterogeneity, pulmonary sarcomatoid adenocarcinoma, sarcomatoid transformation, whole-exome sequencing

## Abstract

**Background:**

Pulmonary sarcomatoid carcinoma (PSC) is a rare and highly aggressive subtype of non-small cell lung cancer characterized by the coexistence of carcinomatous (CA) and sarcomatous (SA) components. Their clonal relationship and genomic divergence remain poorly defined, particularly in adenocarcinoma-derived PSC.

**Methods:**

We performed comparative whole-exome sequencing (WES) on microdissected CA and SA components from six pulmonary sarcomatoid adenocarcinomas (PSAdC). Histopathology and immunohistochemistry were used to characterize epithelial and mesenchymal phenotypes. Somatic mutations were identified using a standard bioinformatics pipeline, followed by gene set enrichment analysis with g:Profiler.

**Results:**

WES identified 133 non-synonymous variants across 34 genes (181 mutational events). Of these, 34.3% were shared, 29.3% were CA-specific, and 36.5% were SA-specific, indicating marked intratumor heterogeneity. Missense mutations predominated (71.4%). Recurrently altered genes included *PCLO, CPS1, FAT1, PDE4DIP*, and *ARID1B*, while canonical drivers *TP53* and *KRAS* showed component-specific distributions. Enrichment analysis revealed over-representation of pathways related to multicellular organism development, chromatin remodeling, transcription factor binding, and DNA double-strand break repair, as well as non-small cell lung cancer signaling. These alterations correlated with epithelial–mesenchymal transition (EMT) features in SA components, including vimentin upregulation and E-cadherin loss.

**Conclusions:**

PSAdC exhibits a monoclonal origin with subsequent genomic diversification between components. Microdissection-based WES reveals pronounced spatial heterogeneity and lineage-specific mutations. Dysregulated chromatin remodeling and DNA repair pathways, together with EMT-associated phenotypes, provide a mechanistic framework for sarcomatoid differentiation and lineage plasticity in this aggressive tumor subtype.

## Introduction

Pulmonary sarcomatoid carcinoma (PSC) is a rare and highly aggressive subtype of non-small cell lung cancer (NSCLC), histologically characterized by the presence of sarcomatous or sarcoma-like components, including spindle and giant cells (1). Clinically, PSC is associated with poor differentiation, rapid disease progression, and limited responsiveness to conventional therapies, resulting in significantly worse survival outcomes compared with other NSCLC subtypes (2, 3). Despite its clinical relevance, the molecular basis of PSC development and its pronounced therapeutic resistance remain incompletely understood.

Although PSC may arise *de novo*, increasing evidence indicates that a subset develops through histological transformation from pre-existing conventional NSCLC (2). Lung adenocarcinoma (LUAD) is the most frequently reported precursor, which we define here as pulmonary sarcomatoid adenocarcinoma (PSAdC). In particular, adenocarcinoma-to-sarcomatoid transformation has been described in LUAD harboring oncogenic driver alterations, such as *EGFR* mutations or *ALK* rearrangements, especially following treatment with tyrosine kinase inhibitors (TKIs) (4, 5). These findings suggest that sarcomatoid transformation represents a mechanism of acquired resistance, enabling tumor cells to escape oncogene dependence and adapt under therapeutic pressure (6, 7).

At the cellular level, this transition is closely linked to epithelial–mesenchymal transition (EMT), characterized by the loss of epithelial features (e.g., E-cadherin) and acquisition of mesenchymal traits (e.g., vimentin) (8, 9). EMT is associated with increased invasiveness, metastatic potential, and resistance to both targeted therapy and chemotherapy (6, 10). Multiple signaling pathways, including ILK/fibronectin and TGF-β signaling, as well as tumor microenvironmental cues, have been implicated in promoting EMT and cellular plasticity (11–13). However, these models do not fully account for the genetic alterations that accompany and potentially drive sarcomatoid differentiation.

Large-scale cancer genomics efforts, such as *The Cancer Genome Atlas* (TCGA) and other public datasets, have provided comprehensive molecular characterization of NSCLC, including LUAD, and have defined key driver mutations and signaling pathways across patient cohorts (14). However, PSC is underrepresented in these datasets due to its rarity (2, 15). Importantly, bulk sequencing approaches do not distinguish between carcinomatous (CA) and sarcomatous (SA) components within the same tumor (8, 10, 13). As a result, component-specific genomic features and the evolutionary relationship between these histological compartments cannot be resolved using existing public data alone (15, 16).

Consistent with this limitation, most genomic studies of PSC have relied on bulk tumor sequencing (10). Although microdissection-based approaches have been applied in a limited number of cases (13), clear and reproducible component-specific mutational profiles have not been established. This may partly reflect the inclusion of PSCs with mixed histological origins in previous studies, complicating lineage-specific interpretation. Consequently, the clonal relationship between CA and SA components, particularly in adenocarcinoma-derived PSC, remains insufficiently defined.

Therefore, a critical gap remains in defining the clonal relationship and component-specific genomic alterations underlying sarcomatoid transformation in adenocarcinoma-derived PSC. Addressing this gap requires a component-resolved genomic approach in a well-defined cohort.

## Materials and methods

### Case selection and clinical data

Between 2021 and 2025, 11 patients who underwent surgical resection and were diagnosed with PSAdC at the First Affiliated Hospital of Guilin Medical University were initially screened. The study was approved by the Institutional Review Board.

Inclusion criteria were:

1. sarcomatoid carcinoma containing adenocarcinoma components.
2. no prior history of malignancy.
3. provision of informed consent.

Exclusion criteria were:

1. insufficient tissue.
2. inability to clearly delineate CA and SA components.
3. pure undifferentiated carcinoma, pure sarcoma, or other distinct differentiation.
4. concurrent malignancies.
5. lack of consent.

Ultimately, 6 primary PSAdC cases were included. Clinical data collected included sex, age, smoking history, tumor location and size, T stage, lymph node metastasis, distant metastasis, recurrence, and survival.

### Morphology and immunohistochemistry

Formalin-fixed paraffin-embedded (FFPE) blocks were sectioned at 3 μm, stained with hematoxylin and eosin (H&E), and independently reviewed by two senior pathologists. Adenocarcinoma growth patterns (acinar, papillary, micropapillary, and solid) and sarcomatoid morphologies (spindle, giant, and pleomorphic) were assessed according to the 2021 WHO classification (1). Transitional zones between components were documented.

Immunohistochemistry (IHC) was performed, as described (17), using antibodies (Fuzhou Maixin) against AE1/AE3, CK7, TTF-1, E-cadherin, vimentin, SMA, desmin, S100, myogenin, and Ki-67, using the MaxVision™ HRP system (Fuzhou Maixin) with DAB chromogen (**Supplementary Table 8**). Positive and negative controls were included.

Slides were evaluated using an Olympus BX53 microscope (Olympus Corporation, Tokyo, Japan) and digitized using a digital pathology slide scanner (Jiangfeng Information Technology Co., Ltd., Ningbo, China).

### Whole-exome sequencing

Whole-exome sequencing was performed on microdissected tumor components (CA and SA) and matched normal samples. DNA was extracted from archival FFPE tissues and sequenced at BGI using the SureSelectXT Human All Exon V6 kit on the DNBSEQ platform (paired-end 100 bp reads; mean depth ≥ 66 x).

Reads were aligned to the reference genome (GRCh38) using BWA-MEM, and somatic mutations were called using GATK HaplotypeCaller following the GATK Best Practices workflow.

To ensure data quality and reliability, stringent filtering criteria were applied. Specifically, a minimum read depth of 20× and a variant quality score (QUAL) ≥ 30 were required. Variants with a population allele frequency ≥ 0.01 (1%) in public databases including gnomAD and the 1000 Genomes Project were excluded to remove common polymorphisms.

Variant allele fraction (VAF) thresholds were applied differentially: ≥ 0.05 for somatic mutations (to detect low-frequency cancer variants) and ≥ 0.25 for germline variants (to ensure reliable detection of constitutional variants). Non-synonymous variants including missense, nonsense, frameshift, and splice-site mutations were retained, while synonymous variants were excluded.

For variants of particular interest, in silico pathogenicity predictions were incorporated, with a CADD score ≥ 20, SIFT ≤ 0.05, or PolyPhen-2 ≥ 0.909 considered supportive evidence of functional impact. Variant annotation was performed using SnpEff, ClinVar, OMIM, and HGMD.

### Gene set enrichment analysis

Functional enrichment analysis was performed on the list of mutated genes using the g:Profiler web server (version e113_eg59_p19_6be52918; database updated on 20 March 2026; https://biit.cs.ut.ee/gprofiler). The analysis encompassed Gene Ontology (GO) categories - Molecular Function (MF), Biological Process (BP), and Cellular Component (CC) - as well as the KEGG, Reactome, and WikiPathways databases. Homo sapiens was selected as the reference organism, with the default whole-genome background. Statistical significance was assessed using the g:SCS (Set Counts and Sizes) multiple testing correction method, with an adjusted p-value threshold of <0.05.

## Results

### Clinicopathological and immunophenotypic characteristics

Six PSAdC cases were included. Most patients were male (83.3%), aged ≥ 60 years (66.7%), and half had a smoking history. Tumors were predominantly peripheral (83.3%) and solitary (100%), with 50% measuring ≥ 3 cm. Four cases (66.7%) involved the left lung. Lymph node metastases occurred in 66.7% of cases, and recurrence or death occurred in 50% (**Table 1**).

**Table 1:** Clinicopathological characteristics of six PSAdC cases.

Characteristic	Variable	n (%)
Gender	Male	5 (83.3%)
	Female	1 (16.7%)
Age	≥ 60 years old	4 (66.7%)
	<60 years old	2 (33.3%)
Smoking status	Non-smokers	3 (50.0%)
	smokers	3 (50.0%)
Tumor location	Left upper lobe	2 (33.3%)
	Left lower lobe	2 (33.3%)
	Right upper lobe	1 (16.7%)
	Right middle and lower lobes	1 (16.7%)
Tumor location	Peripheral type	5 (83.3%)
	Central type	1 (16.7%)
Number of tumors	1	6(100.0%)
	≥ 2	0 (0.0%)
Maximum tumor diameter	≥ 3cm	3 (50.0%)
	< 3cm	3 (50.0%)
T stage	T1	3 (50.0%)
	T2	2 (33.3%)
	T3	1 (16.7%)
	T4	0 (0.0%)
Lymph node metastasis	Present	4 (66.7%)
	Absent	2 (33.3%)
Distant metastasis	Present	2 (33.3%)
	Absent	4 (66.7%)
Recurrence/Death	Present	3 (50.0%)
	Absent	3 (50.0%)

**Figure 1:**
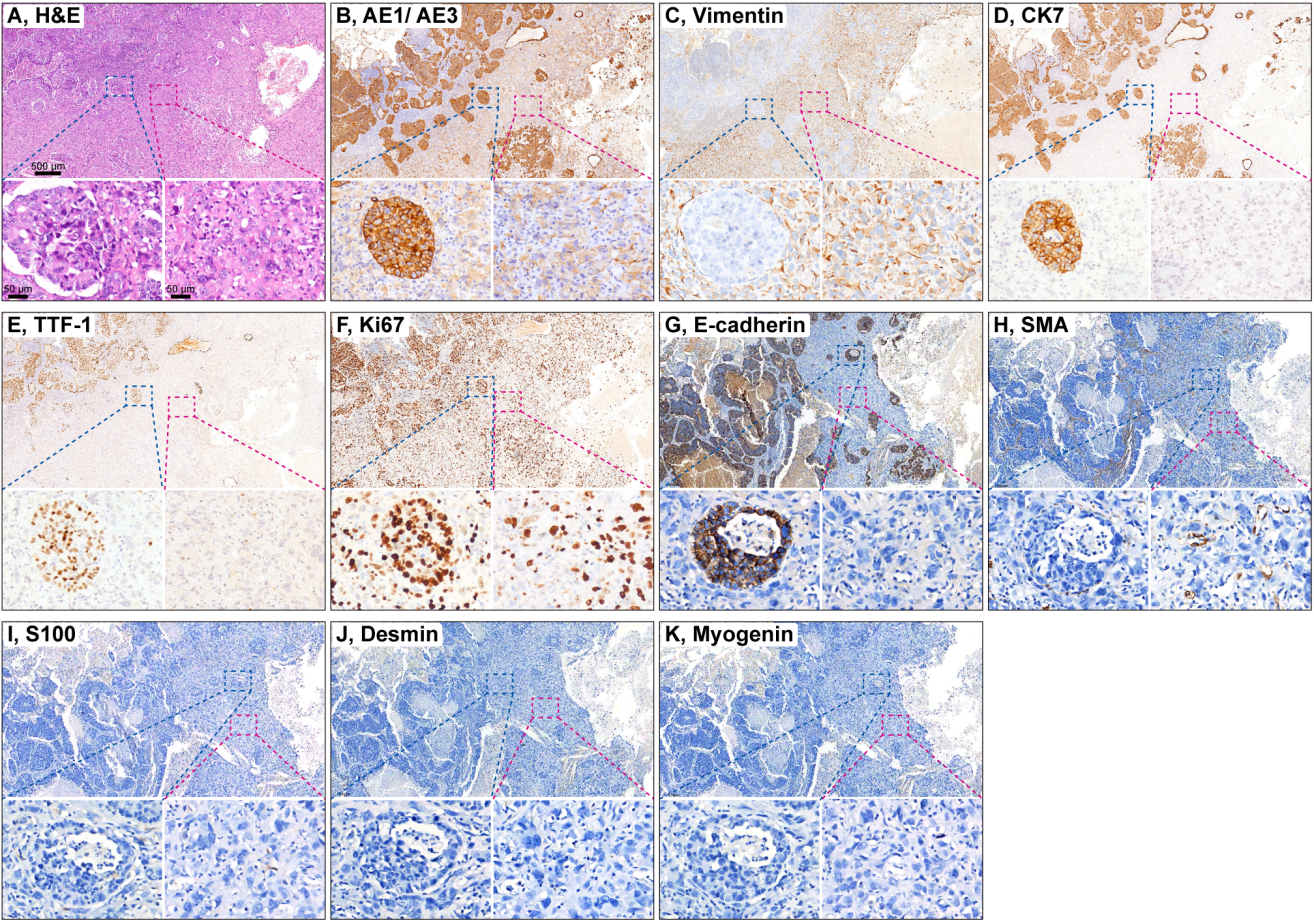
Histopathology and immunophenotypic characterization of PSAdC. AH: Transitional zones between CA and SA. Low-power view (4 X magnification) showing the biphasic architecture and transitional zones between CA and SA. High-power view (40 X magnification) of the transitional region; the left side demonstrates adenocarcinomatous (glandular/acinar) differentiation characteristic of the CA component, while the right side exhibits spindle-cell and pleomorphic morphology typical of the SA component. **(A)** H&E staining. **(B)** AE1/AE3, **(C)** vimentin, **(D)** CK7, **(E)** TTF-1, **(F)** Ki67, **(G)** E-cadherin, **(H)** SMA, **(I)** S100, **(J)** Desmin, **(K)** Myogenin.

CA components displayed glandular architecture with cytologic atypia, whereas SA components consisted of spindle and/or multinucleated giant cells; transitional zones were observed in all cases (**Figure 1A**; **Table 2**). IHC confirmed epithelial marker expression (AE1/AE3, E-cadherin) in CA components with focal vimentin expression, while SA components showed strong vimentin expression with retained AE1/AE3 but loss of E-cadherin, consistent with EMT (**Figures 1B–K**; **Table 3**).

**Table 2:** Histological types and recurrence status of six PSAdC cases.

Case	Histological type		Recurrence/Death
	Adenocarcinoma	Sarcoma	
1	solid pattern 30%	giant cell 70%	death
2	solid pattern 40% acinar pattern 10%	spindle cell 50%	none
3	acinar pattern 40%	giant cell 60%	none
4	acinar pattern 30% papillary pattern 40% micropapillary pattern 10%	spindle cell 20%	recurrence
5	solid pattern 50% cribriform pattern 30%	spindle cell 10% giant cell 10%	none
6	acinar pattern 40% papillary pattern 20% micropapillary pattern 10%	spindle cell 10% giant cell 20%	recurrence

**Table 3:** Immunohistochemical expression in six PSAdC cases.

Immuno histochemical antibodies	Adenocarcinoma		Sarcoma	
	+	-	+	-
AE1/AE3	6 (Strong)	0	6 (faint to weak)	0
CK7	6	0	0	6
TTF-1	6	0	0	6
E-cadherin	6	0	3(faint to weak)	3
Vimentin	2 (faint to weak)	4	6	0
SMA	0	6	1	5
Desmin	0	6	0	6
S100	0	6	0	6
Myogenin	0	6	0	6
Ki67 index	4 (≥50%)	2 (<50%)	3 (≥50%)	3 (<50%)

### Genomic landscape

**Figure 2:**
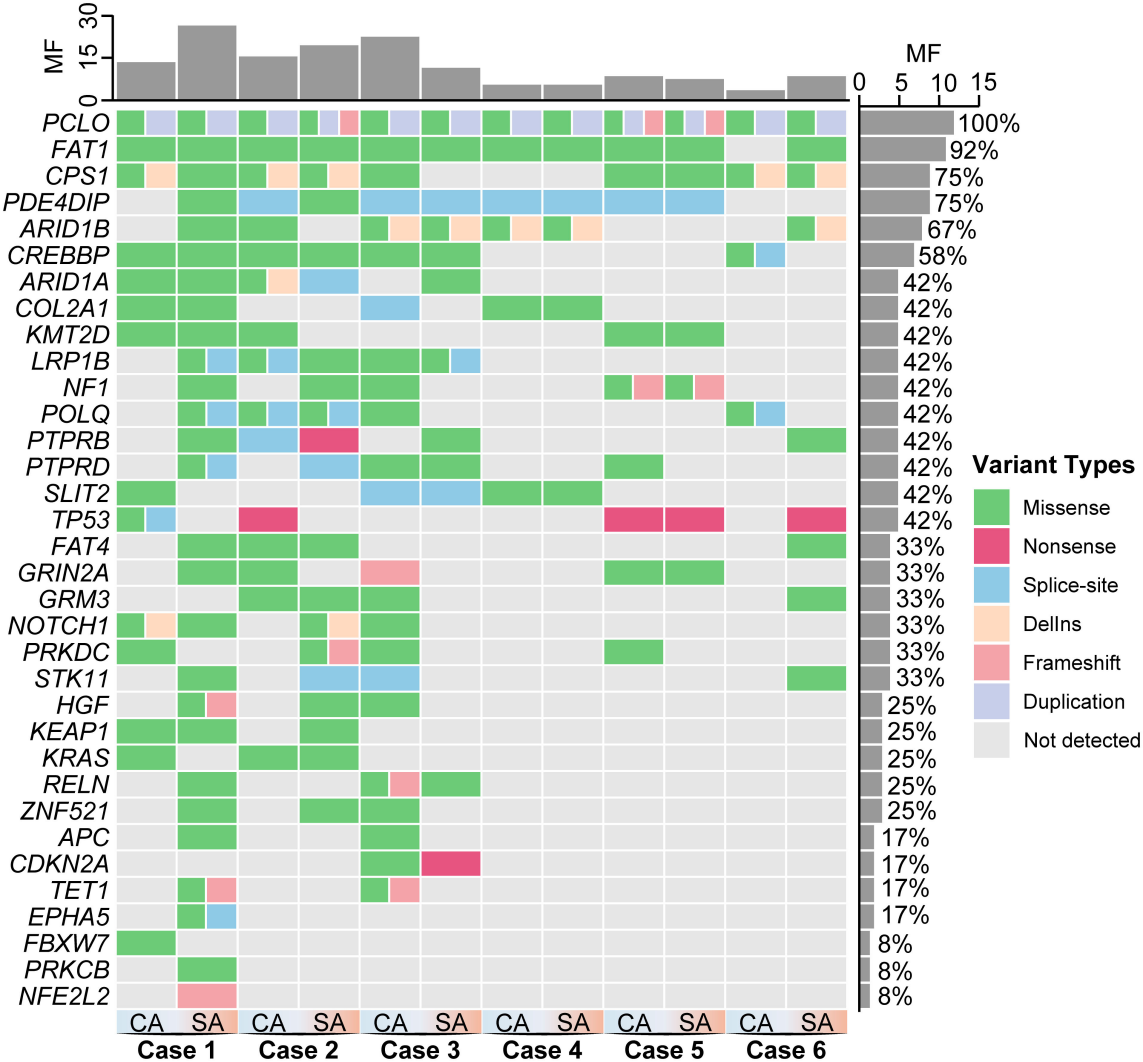
Mutational landscape comparison between carcinomatous and sarcomatoid components in six PSAdC cases. Oncoprint illustrating non-synonymous mutations identified by next-generation sequencing. CA, carcinomatous (adenocarcinoma) component; SA, sarcomatoid component. MF, mutation frequency. Mutations are stratified by variant allele frequency (VAF) and annotated according to AACR/ASCO guidelines.

**Figure 3:**
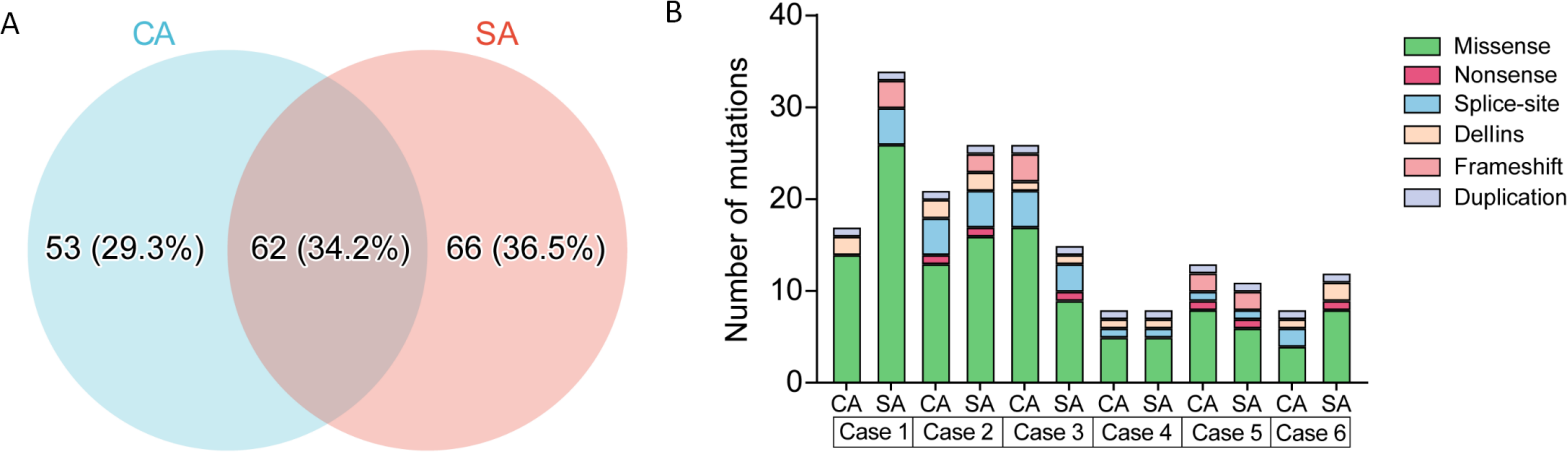
Somatic mutation burden and distribution between carcinomatous (CA) and sarcomatoid (SA) components in PSAdC. **(A)** Distribution of somatic mutation events between CA and SA components. **(B)** Bar graph showing the number of mutations in CA and SA components. No statistically significant differences were observed in mutation frequencies between CA and SA components (p > 0.05).

Whole-exome sequencing identified 133 non-synonymous variants across 34 genes, corresponding to 181 mutational events (**Figure 2**; **Supplementary Tables 1**-**7**). CA components harbored 84 mutations and SA components 97 mutations; shared mutations accounted for 34.3% (62/181), CA-exclusive mutations 29.3% (53/181), and SA-exclusive mutations 36.5% (66/181) (**Figure 3A**). No significant difference in overall mutation frequency was observed between the two components (*P* > 0.05, **Figure 3B**).

Missense variants predominated (71.4%), followed by splice-site alterations (11.3%), frameshifts (9%), insertions/deletions (4.5%), nonsense mutations (3%), and duplications (0.8%). Frequently mutated genes included putative passenger genes such as *PCLO* and *CPS1*, alongside established cancer-associated genes including *FAT1*, *PDE4DIP*, and *ARID1B* (**Figure 2**; **Supplementary Tables 1**–**7**).

Interestingly, classic lung adenocarcinoma driver genes such as *TP53* and *KRAS* exhibited relatively low overall mutation frequencies. Rather than acting as ubiquitous early events, these drivers frequently appeared as component-specific alterations restricted to either the CA or SA regions. This spatial distribution highlights pronounced subclonal divergence and substantial intratumor heterogeneity during sarcomatoid differentiation.

The mutational landscape also involved genes linked to RAS/MAPK, Wnt, chromatin remodeling, Hippo, and oxidative stress response pathways. Notably, every case harbored at least one alteration in these networks, which are known to facilitate mesenchymal transition. Pathway alterations within SA components were further associated with strong vimentin expression and loss of E-cadherin at the protein level.

### Gene set enrichment analysis

**Figure 4:**
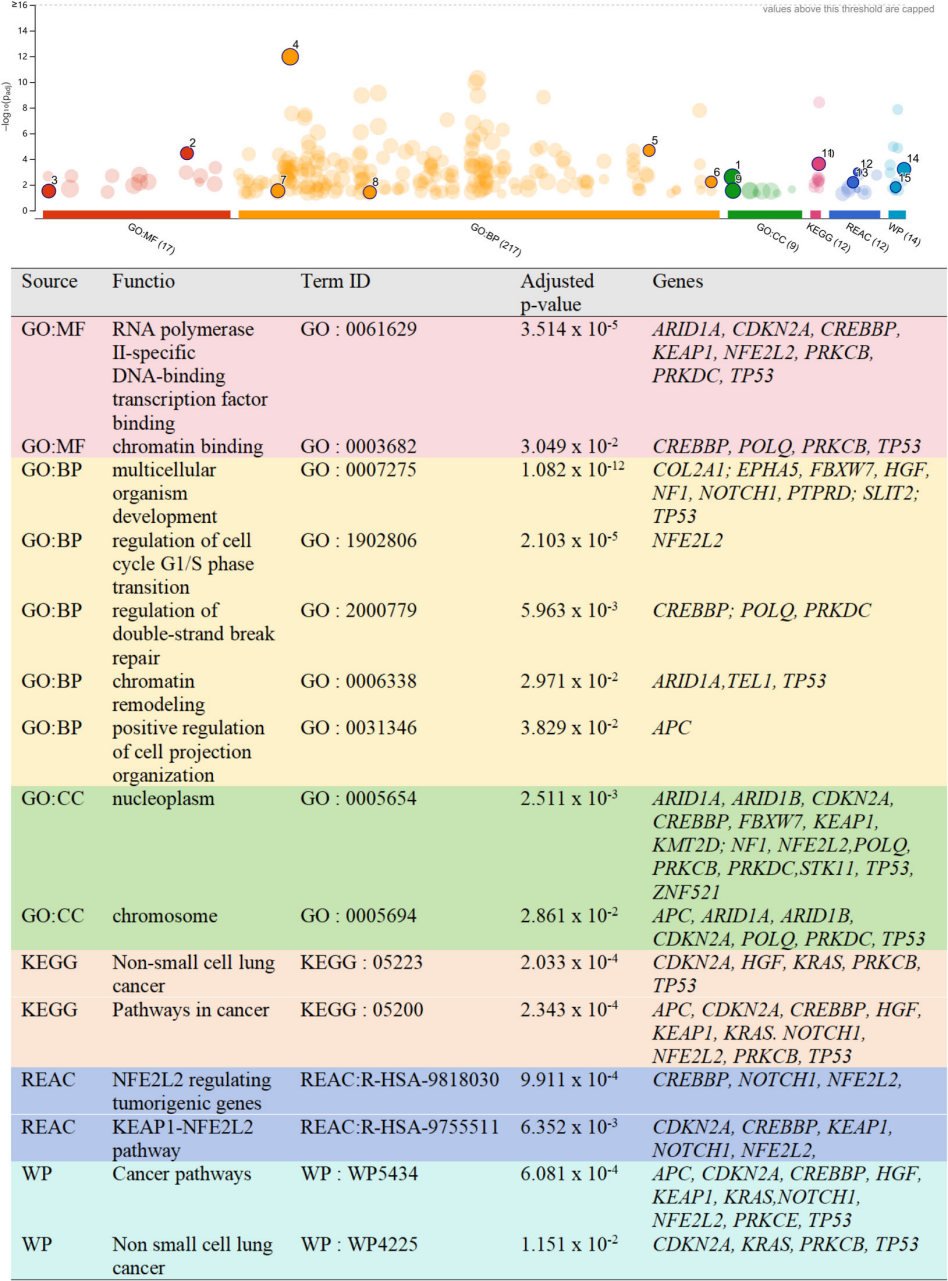
Gene ontology and pathway enrichment analysis of mutated genes in PSAdC. Enrichment analysis of mutated genes from carcinomatous and sarcomatous components of PSAdC was performed using g:Profiler. Significantly enriched terms (adjusted p < 0.05) across GO (MF, BP, CC), KEGG, Reactome, and WikiPathways are shown. Prominent terms include multicellular organism development, chromatin remodeling, transcriptional regulation, and non-small cell lung cancer pathways.

Gene Ontology and pathway enrichment analyses were performed on the altered genes identified in PSAdC samples, with significantly enriched terms summarized in **Figure 4**.

The most prominently enriched Biological Process was multicellular organism development, involving genes such as *COL2A1*, *EPHA5*, *FBXW7*, *HGF*, *NF1*, *NOTCH1*, *PTPRD*, *SLIT2*, and *TP53*. Additional enriched processes included regulation of the G1/S phase transition of the cell cycle, regulation of double-strand break repair, chromatin remodeling, and positive regulation of cell projection organization.

Significant enrichment was also observed for RNA polymerase II-specific DNA-binding transcription factor binding and chromatin binding (Molecular Function), as well as nucleoplasm and chromosome (Cellular Component). Pathway analysis further highlighted enrichment in NSCLC, pathways in cancer, the KEAP1 - NFE2L2 pathway, and WikiPathways cancer-related pathways.

Collectively, these findings indicate dysregulation of developmental processes, transcriptional regulation, chromatin organization, and core oncogenic signaling pathways in pulmonary sarcomatoid carcinoma, which are closely associated with mesenchymal transition and sarcomatoid differentiation.

## Discussion

PSC is characterized by a distinctive biphasic morphology comprising carcinomatous (CA) and sarcomatous (SA) components, along with an aggressive clinical course (3). Many PSCs are thought to arise from LUAD through sarcomatoid transdifferentiation, often driven by therapeutic or microenvironmental selective pressures (4, 6). However, prior genomic studies have predominantly relied on bulk tumor sequencing, leaving component-specific alterations in PSC, particularly those with an adenocarcinoma component, largely unexplored (10). By performing whole-exome sequencing on microdissected CA and SA components, the present study provides direct insights into this critical but understudied aspect of PSC biology.

Our data support a monoclonal origin of the tumor, followed by extensive mutational diversification. Although 34.3% of variants were shared between components, forming a common genomic trunk, the majority (65.7%) were spatially restricted, highlighting pronounced intratumor heterogeneity. Notably, canonical driver mutations such as *TP53* and *KRAS* (11, 18) were not uniformly distributed but frequently emerged as component-specific events. This divergent pattern underscores the subclonal evolution inherent to PSAdC pathogenesis and progression, rather than merely reflecting limited sample size.

EMT is a central mechanism driving sarcomatoid differentiation in PSC (9). In this study, Gene Ontology enrichment analysis revealed significant over-representation of processes related to multicellular organism development, chromatin remodeling, double-strand break repair, and transcriptional regulation, including RNA polymerase II-specific DNA-binding transcription factor binding and chromatin binding. These convergent signatures support a model in which coordinated epigenetic and transcriptional reprogramming underpins the observed mutational landscape and facilitates phenotypic transition (10, 13).

Recurrent alterations in SWI/SNF complex subunits (ARID1A and ARID1B) and epigenetic regulators such as CREBBP indicate disrupted chromatin accessibility and enhancer reprogramming, thereby promoting activation of mesenchymal transcriptional programs and repression of epithelial identity (19–21). Variants in POLQ, a key mediator of error-prone double-strand break repair, may further exacerbate genomic instability and enhance cellular plasticity (22). Alterations involving KRAS, NF1, and TP53 additionally drive RAS/MAPK activation, Hippo pathway dysregulation, and loss of cell adhesion, collectively establishing a permissive molecular framework for EMT induction, sarcomatoid transformation, and the acquisition of spindle-cell morphology (23, 24).

The component-specific mutations identified here also carry potential clinical implications. Distinct driver alterations restricted to either the CA or SA component suggest that single-region biopsies may underestimate the full mutational spectrum of PSAdC, potentially contributing to discordant therapeutic responses observed in practice (25, 26). For example, a *KRAS* mutation confined to the CA component could result in incomplete response if the SA population persists, whereas SA-restricted alterations in *NF1* or *ARID1A* may represent unique vulnerabilities in the more aggressive mesenchymal compartment (27). These findings highlight the value of multi-region sampling or component-aware molecular profiling to inform precision treatment strategies in PSAdC.

Furthermore, the high tumor mutational burden and frequent alterations in immune-related pathways observed in PSC suggest potential benefits from immunotherapy-based regimens, particularly in cases with component-specific drivers (26–28). Recent real-world analyses indicate that targeted therapies against actionable alterations (such as *MET* exon 14 skipping or *KRAS* G12C) may produce differential responses depending on the dominant histological component, underscoring the need for comprehensive genomic profiling (29–31). Collectively, these findings support the integration of component-specific molecular characterization into routine clinical decision-making to optimize therapeutic outcomes in this aggressive subtype.

Despite these insights, several limitations should be acknowledged. The small cohort size restricts the generalizability of mutation frequencies. Given the extreme rarity of strictly defined PSAdC, public databases such as TCGA lack component-resolved sequencing data, precluding direct comparative validation. Consequently, recurrent alterations in genes such as *PCLO, FAT1*, and *FAT4* should be interpreted primarily as indicators of spatial heterogeneity rather than established population-level drivers. Furthermore, the use of archival FFPE tissues and moderate sequencing depth limited our capacity to perform functional validation or comprehensive copy number analysis.

Future multi-institutional studies incorporating multi-omics approaches, including component-specific transcriptomics and epigenomics, will be essential to validate these observations and to elucidate the precise molecular switches governing EMT-driven sarcomatoid transformation in PSC.

## Conclusion

This study presents a microdissection-based, component-resolved genomic analysis of pulmonary sarcomatoid carcinoma arising from lung adenocarcinoma. Whole-exome sequencing and Gene Ontology enrichment reveal pronounced intratumor heterogeneity and genomic divergence between carcinomatous and sarcomatous components. Alterations in SWI/SNF complex subunits (ARID1A, ARID1B), epigenetic regulators (CREBBP), and pathways involving KRAS, NF1, and TP53 converge on processes related to chromatin remodeling, transcriptional regulation, DNA repair, and epithelial - mesenchymal transition, collectively facilitating sarcomatoid differentiation.

These findings highlight the limitations of bulk sequencing and single-region sampling, emphasizing the need for component-specific molecular profiling. Despite the small cohort, this work provides a lineage-resolved framework for understanding sarcomatoid transformation and identifies candidate alterations linked to the aggressive sarcomatous phenotype, with potential implications for precision therapy.

## Data Availability

The datasets presented in this study can be found in online repositories. The names of the repository/repositories and accession number(s) can be found in the article/**Supplementary Material**.

## References

[1] Pelosi G Sonzogni A De Pas T Galetta D Veronesi G Spaggiari L . Review article: pulmonary sarcomatoid carcinomas: a practical overview. Int J Surg Pathol. (2010) 18:103–20. doi: 10.1177/1066896908330049. PMID: 19124452

[2] Li X Wu D Liu H Chen J . Pulmonary sarcomatoid carcinoma: progress, treatment and expectations. Ther Adv Med Oncol. (2020) 12:1758835920950207. doi: 10.1177/1758835920950207. PMID: 32922522 PMC7450456

[3] Martin LW Correa AM Ordonez NG Roth JA Swisher SG Vaporciyan AA . Sarcomatoid carcinoma of the lung: a predictor of poor prognosis. Ann Thorac Surg. (2007) 84:973–80. doi: 10.1016/j.athoracsur.2007.03.099. PMID: 17720411

[4] Pang L Zhuang W Huang Y Liao J Yang M Zhang L . Rare transformation from lung adenocarcinoma to sarcomatoid carcinoma mediates resistance to inhibitors targeting different driver oncogenes. J Natl Cancer Cent. (2025) 5:75–81. doi: 10.1016/j.jncc.2024.12.005. PMID: 40040879 PMC11873652

[5] Hsieh M Lin M Lee Y . Lung adenocarcinoma with sarcomatoid transformation after tyrosine kinase inhibitor treatment and chemotherapy. Lung Cancer. (2019) 137:76–84. doi: 10.1016/j.lungcan.2019.08.029. PMID: 31561203

[6] Wei Y Wang L Jin Z Jia Q Brcic L Akaba T . Biological characteristics and clinical treatment of pulmonary sarcomatoid carcinoma: a narrative review. Transl Lung Cancer Res. (2024) 13:635–53. doi: 10.21037/tlcr-24-127. PMID: 38601447 PMC11002509

[7] Toyokawa G Bersani F Bironzo P Picca F Tabbò F Haratake N . Tumor plasticity and therapeutic resistance in oncogene-addicted non-small cell lung cancer: from preclinical observations to clinical implications. Crit Rev Oncol Hematol. (2023) 184:103966. doi: 10.1016/j.critrevonc.2023.103966. PMID: 36925092

[8] Zhu J Xiong Y Ma S Wei J Chen J Wang W . Single-nucleus RNA sequencing reveals the distinct heterogeneity of primary pulmonary sarcomas (PPS) and pulmonary sarcomatoid carcinoma (PSC). Clin Transl Med. (2026) 16:e70566. doi: 10.1002/ctm2.70566. PMID: 41469334 PMC12753329

[9] Guarino M . Epithelial-mesenchymal transition as a pathogenetic mechanism of sarcomatoid carcinoma and carcinosarcoma. J Clin Pract Res. (2025) 47:345–54. doi: 10.14744/cpr.2025.44690. PMID: 41257063 PMC12478579

[10] Yang Z Xu J Li L Li R Wang Y Tian Y . Integrated molecular characterization reveals potential therapeutic strategies for pulmonary sarcomatoid carcinoma. Nat Commun. (2020) 11:4878. doi: 10.1038/s41467-020-18702-3. PMID: 32985499 PMC7522294

[11] Shimizu S Sakai K Chikugo T Satou T Shiraishi N Mitsudomi T . Integrin-linked kinase pathway in heterogeneous pulmonary sarcomatoid carcinoma. Oncol Lett. (2021) 21:320. doi: 10.3892/ol.2021.12582. PMID: 33692852 PMC7933776

[12] Seth S Chen R Liu Y Fujimoto J Hong L Reuben A . Integrative genomic and transcriptomic profiling of pulmonary sarcomatoid carcinoma identifies molecular subtypes associated with distinct immune features and clinical outcomes. Cancer Innov. (2024) 3:e112. doi: 10.1002/cai2.112. PMID: 38947760 PMC11212327

[13] Kwon HJ Lee S Han YB Lee J Kwon S Kim H . Genomic landscape of pulmonary sarcomatoid carcinoma. Cancer Res Treat. (2024) 56:442–54. doi: 10.4143/crt.2023.764. PMID: 37973906 PMC11016656

[14] Cancer Genome Atlas Research Network . Comprehensive molecular profiling of lung adenocarcinoma. Nature. (2014) 511:543–50. doi: 10.1038/nature13385. PMID: 25079552 PMC4231481

[15] Zhou F Huang Y Cai W Li J Su C Ren S . The genomic and immunologic profiles of pure pulmonary sarcomatoid carcinoma in Chinese patients. Lung Cancer. (2021) 153:66–72. doi: 10.1016/j.lungcan.2021.01.006. PMID: 33454519

[16] Nakagomi T Goto T Hirotsu Y Shikata D Yokoyama Y Higuchi R . New therapeutic targets for pulmonary sarcomatoid carcinomas based on their genomic and phylogenetic profiles. Oncotarget. (2018) 9:10635–49. doi: 10.18632/oncotarget.24365. PMID: 29535832 PMC5828205

[17] Wu H Yang J Yuan L Tan Z Zhang X Hambly BD . IL-38 promotes the development of prostate cancer. Front Immunol. (2024) 15:1384416. doi: 10.3389/fimmu.2024.1384416. PMID: 38779687 PMC11109393

[18] Lococo F Gandolfi G Rossi G Pinto C Rapicetta C Cavazza A . Deep sequencing analysis reveals that KRAS mutation is a marker of poor prognosis in patients with pulmonary sarcomatoid carcinoma. J Thorac Oncol. (2016) 11:1282–92. doi: 10.1016/j.jtho.2016.04.020. PMID: 27156442

[19] Jin F Yang Z Shao J Tao J Reißfelder C Loges S . ARID1A mutations in lung cancer: biology, prognostic role, and therapeutic implications. Trends Mol Med. (2023) 29:646–58. doi: 10.1016/j.molmed.2023.04.005. PMID: 37179132

[20] Kelso T Porter DK Amaral ML Shokhirev MN Benner C Hargreaves DC . Chromatin accessibility underlies synthetic lethality of SWI/SNF subunits in ARID1A-mutant cancers. Elife. (2017) 6. doi: 10.7554/elife.30506. PMID: 28967863 PMC5643100

[21] Shang XY Shi Y He DD Wang L Luo Q Deng CH . ARID1A deficiency weakens BRG1-RAD21 interaction that jeopardizes chromatin compactness and drives liver cancer cell metastasis. Cell Death Dis. (2021) 12:990. doi: 10.1038/s41419-021-04291-6. PMID: 34689165 PMC8542038

[22] Moyret-Lalle C Prodhomme MK Burlet D Kashiwagi A Petrilli V Puisieux A . Role of EMT in the DNA damage response, double-strand break repair pathway choice and its implications in cancer treatment. Cancer Sci. (2022) 113:2214–23. doi: 10.1111/cas.15389. PMID: 35534984 PMC9277259

[23] Mehrad M Roy S LaFramboise WA Petrosko P Miller C Incharoen P . KRAS mutation is predictive of outcome in patients with pulmonary sarcomatoid carcinoma. Histopathology. (2018) 73:207–14. doi: 10.1111/his.13505. PMID: 29489023 PMC7393997

[24] Ullah A Ahmed A Yasinzai AQK Lee KT Khan I Asif B . Demographics and clinicopathologic profile of pulmonary sarcomatoid carcinoma with survival analysis and genomic landscape. Cancers (Basel). (2023) 15. doi: 10.3390/cancers15092469. PMID: 37173936 PMC10177027

[25] Liu X Wang F Xu C Chen X Hou X Li Q . Genomic origin and intratumor heterogeneity revealed by sequencing on carcinomatous and sarcomatous components of pulmonary sarcomatoid carcinoma. Oncogene. (2021) 40:821–32. doi: 10.1038/s41388-020-01573-9. PMID: 33273725

[26] Schrock AB Li SD Frampton GM Suh J Braun E Mehra R . Pulmonary sarcomatoid carcinomas commonly harbor either potentially targetable genomic alterations or high tumor mutational burden as observed by comprehensive genomic profiling. J Thorac Oncol. (2017) 12:932–42. doi: 10.1016/j.jtho.2017.03.005. PMID: 28315738

[27] Hong L Di Federico A Liu B Cooper AJ Alessi JV Clark P . Distinct clinicogenomic features and immunotherapy associations in pulmonary sarcomatoid carcinoma: a multicenter retrospective study. J Thorac Oncol. (2026) 20:1763–77. doi: 10.1016/j.jtho.2025.07.121. PMID: 40716572 PMC12466414

[28] Inomata M Tsuda T Ichikawa T Matsumoto M Mizushima I Azechi K . Efficacy of immune checkpoint inhibitor therapy in patients with pulmonary sarcomatoid carcinoma in clinical practice. Thorac Cancer. (2023) 14:1618–23. doi: 10.1111/1759-7714.14907. PMID: 37101081 PMC10260484

[29] Tsuda T Ichikawa T Matsumoto M Mizusihima I Azechi K Takata N . An observational study on the efficacy of targeted therapy for pulmonary sarcomatoid carcinoma. Discov Oncol. (2024) 15:382. doi: 10.1007/s12672-024-01046-5. PMID: 39207576 PMC11362448

[30] Wang Q Guo H Wang Y He T Diao M Wu Y . The pathogenesis and therapeutic management of rare pulmonary sarcomatoid carcinoma: a narrative review. Transl Lung Cancer Res. (2025) 14:5137–58. doi: 10.21037/tlcr-2025-886. PMID: 41367587 PMC12683482

[31] Chang CL Hsieh MS Shih JY Lee YH Liao WY Hsu CL . Real-world treatment patterns and outcomes among patients with advanced non-small-cell lung cancer with spindle cell and/or giant cell carcinoma. Ther Adv Med Oncol. (2022) 14:17588359221133889. doi: 10.1177/17588359221133889. PMID: 36324732 PMC9618761

