# Supplementary material for: Genomic characterization of pulmonary sarcomatoid adenocarcinoma: a paired whole-exome sequencing study of carcinomatous and sarcomatous components
Source: Front Oncol. 2026 Apr 22;16:1796428. doi: 10.3389/fonc.2026.1796428 (PMC13143603; doi:10.3389/fonc.2026.1796428)
Supplement: Supplementary file 1 [file Table1.docx]

**supplementary tables：**

| **Supplementary** **Table 1. Somatic variant profile of Patient 1 tumor tissue** | | | | | |
| --- | --- | --- | --- | --- | --- |
| **Gene** | **Variant Types** | **Variant** | **Chromosomal Location (GRCh38)** | **VAF** | |
|  |  |  |  | **CA** | **SA** |
| *APC* | Missense | ENST00000508376:p.Pro2158Ser/c.6472C>T | chr5:112842066 | - | 25% |
| *ARID1A* | Missense | ENST00000324856:p.Gly1556Asp/c.4667G>A | chr1:26774894 | 15% | - |
| *ARID1A* | Missense | ENST00000324856:p.Arg1918Gln/c.5753G>A | chr1:26779651 | - | 33% |
| *ARID1B* | Missense | ENST00000636930:p.Arg1198Cys/c.3592C>T | chr6:157184357 | - | 33% |
| *COL2A1* | Missense | ENST00000380518:p.Lys44Asn/c.132G>T | chr12:48000079 | 22% | - |
| *COL2A1* | Missense | ENST00000380518:p.Ala1199Thr/c.3595C>T | chr12:47975965 | - | 29% |
| *CPS1* | DelIns | ENST00000430249:ACCdelinsGCT | chr2:210591913 - 210591915 | 45% | 30% |
| *CPS1* | DelIns | ENST00000430249:p.Ile5_Lys6insPhe/c.15_16insTTC | chr2:210556728 - 210556730 | 32% | - |
| *CREBBP* | Missense | ENST00000262367:p.Met2032Ile/c.6096C>T | chr16:3728951 | 25% | - |
| *CREBBP* | Missense | ENST00000262367:p.Gly194Val/c.581G>T | chr16:3850514 | - | 33% |
| *EPHA5* | Splice site | ENST00000622150:p.Val304Val/c.912C>A | chr4:65495542 | - | 29% |
| *EPHA5* | Missense | ENST00000622150:p.Ser451Ile/c.1352C>T | chr4:65490427 | - | 29% |
| *FAT1* | Missense | ENST00000614102:p.Ala4553Gly/c.13658C>G | chr4:186588707 | 53%% | - |
| *FAT1* | Missense | ENST00000614102:p.Gly2628Cys/c.7882G>T | chr4:186618710 | - | 29% |
| *FAT4* | Missense | ENST00000394329:p.Ala3822Ser/c.11464G>T | chr4:125452480 | - | 29% |
| *FBXW7* | Missense | ENST00000281708:p.Ala204Thr/c.610G>A | chr4:152347046 | 32% | - |
| *GRIN2A* | Missense | ENST00000330684:p.Val1000Met/c.2998C>T | chr16:9764546 | - | 29% |
| *HGF* | Missense | ENST00000222390:p.Gly229Val/c.686G>T | chr7:81745060 | - | 29% |
| *HGF* | Frameshift | ENST00000222390:p.Thr490fs/c.1468delA | chr7:81710219 | - | 33% |
| *KEAP1* | Missense | ENST00000437147:p.Glu351Lys/c.1051G>A | chr19:10491617 | 22% | - |
| *KEAP1* | Missense | ENST00000171111:p.Arg204Gln/c.611G>A | chr19:10499423 | - | 29% |
| *KMT2D* | Splice site | ENST00000301067:c.5645-4A>G | chr12:49042882 | 33% | - |
| *KMT2D* | Missense | ENST00000301067:p.Arg1311Ile/c.3932G>T | chr12:49049193 | - | 20% |
| *KRAS* | Missense | ENST00000256078:p.Gly12Cys/c.34G>T | chr12:25245351 | 28% | - |
| *LRP1B* | Missense | ENST00000442974:p.Pro163Ser/c.487C>T | chr2:140269335 | - | 25% |
| *LRP1B* | Splice site | ENST00000389484:c.4170-3delT | chr2:140868265 | - | 23% |
| *NF1* | Missense | ENST00000579081:p.Arg31Ser/c.91C>A | chr17:31095298 | - | 33% |
| *NOTCH1* | Missense | ENST00000277541:p.Ala729Thr/c.2185G>A | chr9:136514532 | 29% | - |
| *NOTCH1* | Missense | ENST00000277541:p.Asn2321Ser/c.6962A>G | chr9:136496777 | - | 40% |
| *NOTCH1* | DelIns | ENST00000277541:p.Pro2415del/c.7244_7246delCAC | chr9:136496492 | 22% | - |
| *NFE2L2* | Frameshift | ENST00000397062:p.Asn546fs/c.1637delA | chr2:177230965 | - | 33% |
| *PCLO* | Missense | ENST00000333891:p.Pro469Thr/c.1405C>A | chr7:83155236 | - | 40% |
| *PCLO* | Duplication | ENST00000333891:p.Asp2926dup/c.8778_8780insTGA | chr7:82952172 | 78% | 71% |
| *PDE4DIP* | Missense | ENST00000585156:p.Glu1752Asp/c.5256A>T | chr1:149009712 | - | 40% |
| *POLQ* | Missense | ENST00000621776:p.Cys832Tyr/c.2495G>A | chr3:121498540 | - | 40% |
| *POLQ* | Missense | ENST00000621776:p.Ala2341Val/c.7022C>T | chr3:121472091 | - | 25% |
| *POLQ* | Splice site | ENST00000621776:c.6179-6_6179-5delTT | chr3:121483586 | - | 50% |
| *PRKCB* | Missense | ENST00000303531:p.Glu591Lys/c.1771G>A | chr16:24191138 | - | 33% |
| *PRKDC* | Missense | ENST00000314191:p.Arg3282His/c.9845G>A | chr8:47803383 | 15% | - |
| *PTPRB* | Missense | ENST00000334414:p.Arg1933Gln/c.5798G>A | chr12:70538995 | - | 33% |
| *PTPRD* | Missense | ENST00000356435:p.Val1511Met/c.4531G>A | chr9:8376066 | - | 25% |
| *PTPRD* | Splice site | ENST00000356435:c.551-5C>T | chr9:8526649 | - | 50% |
| *RELN* | Missense | ENST00000424685:p.Trp652Ser/c.1955G>C | chr7:103650321 | - | 29% |
| *SLIT2* | Missense | ENST00000273739:p.Gly1319Asp/c.3956G>A | chr4:20616979 | 25% | - |
| *STK11* | Missense | ENST00000326873:p.Ala406Gly/c.1217C>G | chr19:1226562 | - | 33% |
| *TET1* | Frameshift | ENST00000373644:p.Pro731fs/c.2192delC | chr10:68644918 | - | 33% |
| *TP53* | Splice site | ENST00000413465:c.783-6_783-5delCT | chr17:7662018 | 25% | - |
| *ZNF521* | Missense | ENST00000361524:p.Ser233Phe/c.698C>T | chr18:25227220 | - | 33% |

| **Supplementary** **Table 2. Somatic variant profile of Patient 2 tumor tissue** | | | | | |
| --- | --- | --- | --- | --- | --- |
| **Gene** | **Variant Types** | **Variant** | **Chromosomal Location (GRCh38)** | **VAF** | |
|  |  |  |  | **CA** | **SA** |
| *ARID1A* | Splice site | ENST00000324856:c.3866+6C>A | chr1:26773502 | - | 22% |
| *ARID1A* | DelIns | ENST00000324856:p.Gly190del/c.567_569delCGG | chr1:26696959 - 26696961 | 22% | - |
| *ARID1B* | Missense | ENST00000636930:p.Glu439Lys/c.1315G>A | chr6:156779244 | 25% | - |
| *CPS1* | DelIns | ENST00000430249:p.Thr350Ala/c.1048ACCdelinsGCT | chr2:210591913 - 210591915 | 100% | 100% |
| *CPS1* | DelIns | ENST00000430249:p.Ile5_Lys6insPhe/c.15_16insTTC | chr2:210556728 | 100% | 100% |
| *CREBBP* | Missense | ENST00000262367:p.Pro2311Ser/c.6931C>T | chr16:3728116 | 25% | - |
| *CREBBP* | Missense | ENST00000262367:p.Gly1469Arg/c.4405G>A | chr16:3736805 | - | 29% |
| *FAT1* | Missense | ENST00000614102:p.Gln2800Leu/c.8399A>T | chr4:186618193 | 25% | - |
| *FAT1* | Missense | ENST00000614102:p.Gly3986Arg/c.11956C>T | chr4:186600051 | - | 22% |
| *FAT1* | Missense | ENST00000614102:p.Glu3988Lys/c.11962C>T | chr4:186600045 | - | 18% |
| *FAT4* | Missense | ENST00000394329:p.Asp284Asn/c.850G>A | chr4:125317261 | 33% | - |
| *FAT4* | Missense | ENST00000394329:p.Gly3932Arg/c.11794G>A | chr4:125452810 | - | 25% |
| *GRIN2A* | Missense | ENST00000330684:p.His1371Tyr/c.4111G>A | chr16:9763433 | 14% | - |
| *GRM3* | Missense | ENST00000454217:p.Pro10Ser/c.28C>T | chr7:86644816 | 29% | 67% |
| *HGF* | Missense | ENST00000222390:p.Leu19Phe/c.55C>T | chr7:81769917 | - | 61% |
| *KEAP1* | Missense | ENST00000171111:p.Val594Met/c.1780G>A | chr19:10486747 | - | 25% |
| *KMT2D* | Missense | ENST00000301067:p.Gln2356His/c.7068G>T | chr12:49040702 | 40% | - |
| *KRAS* | Missense | ENST00000256078:p.Gly12Cys/c.34G>T | chr12:25245351 | 54% | 37% |
| *LRP1B* | Missense | ENST00000389484:p.Cys2951Ser/c.8852G>C | chr2:140495747 | - | 29% |
| *LRP1B* | Splice site | ENST00000389484:c.4170-3delT | chr2:140868265 | 36% | - |
| *NF1* | Missense | ENST00000358273:p.Ile838Ser/c.2513T>G | chr17:31229128 | - | 33% |
| *NOTCH1* | Missense | ENST00000277541:p.Asp297Asn/c.889G>A | chr9:136518801 | - | 29% |
| *NOTCH1* | DelIns | ENST00000277541:p.Pro401fs/c.1189_1201insGGCAGGTGCAGAT | chr9:136518190 | - | 33% |
| *PCLO* | Missense | ENST00000333891:p.Pro2082Leu/c.6245C>T | chr7:82954708 | 40% | - |
| *PCLO* | Missense | ENST00000333891:p.Gly3018Glu/c.9053G>A | chr7:82951900 | - | 25% |
| *PCLO* | Duplication | ENST00000333891:p.Asp2926dup/c.8778_8780insTGA | chr7:82952172 | 63% | 78% |
| *PCLO* | Frameshift | ENST00000333891:p.Lys1394fs/c.4181delA | chr7:82956771 | - | 22% |
| *PDE4DIP* | Splice site | ENST00000585156:c.4178-6A>T | chr1:149003608 | 33% | - |
| *PDE4DIP* | Missense | ENST00000585156:p.Arg567Leu/c.1700G>T | chr1:148965578 | - | 22% |
| *POLQ* | Splice site | ENST00000621776:c.6179-5dupT | chr3:121483586 | 23% | 33% |
| *PRKDC* | Frameshift | ENST00000314191:p.Asp2376fs/c.7128_7129delCA | chr8:47849379 - 47849380 | - | 20% |
| *PTPRB* | Splice site | ENST00000334414:c.980-7C>T | chr12:70596334 | 33% | - |
| *PTPRB* | Nonsense | ENST00000334414:p.Gln2071*/c.6211C>T | chr12:70534645 | - | 21% |
| *PTPRD* | Splice site | ENST00000356435:c.551-5C>T | chr9:8526649 | - | 14% |
| *STK11* | Splice site | ENST00000326873:c.375-1C>T | chr19:1219323 | - | 25% |
| *TP53* | Nonsense | ENST00000635293:p.Gly227*/c.679G>T | chr17:7673824 | 44% | - |
| *ZNF521* | Missense | ENST00000361524:p.Arg602Gly/c.1804A>G | chr18:25226114 | - | 13% |

| **Supplementary** **Table 3. Somatic variant profile of Patient 3 tumor tissue** | | | | | |
| --- | --- | --- | --- | --- | --- |
| **Gene** | **Variant Types** | **Variant** | **Chromosomal Location (GRCh38)** | **VAF** | |
|  |  |  |  | **CA** | **SA** |
| *ARID1A* | Missense | ENST00000324856:p.Asp1893Glu/c.5679C>G | chr1:26779577 | - | 65% |
| *ARID1B* | Missense | ENST00000636930:p.Met570Ile/c.1710G>A | chr6:156829394 | 29% | - |
| *ARID1B* | DelIns | ENST00000636930:p.Ala460del/c.1379_1381delCGG | chr6:156779296 - 156779299 | 25% | - |
| *ARID1B* | DelIns | ENST00000636930:p.Gly328del/c.983_985delGAG | chr6:156778889 - 156778891 | - | 13% |
| *CDKN2A* | Nonsense | ENST00000498124:p.Trp15*/c.45G>A | chr9:21974783 | - | 33% |
| *CDKN2A* | Missense | ENST00000498124:p.Gly122Cys/c.364G>T | chr9:21970995 | 40% | - |
| *COL2A1* | Splice site | ENST00000380518:c.817-2A>G | chr12:47994049 | 33% | - |
| *CPS1* | Missense | ENST00000430249:p.Arg244Gly/c.730C>G | chr2:210590106 | 40% | - |
| *CREBBP* | Missense | ENST00000262367:p.Pro795Ser/c.2383G>A | chr16:3773831 | 50% | - |
| *CREBBP* | Missense | ENST00000262367:p.Lys1627Asn/c.4881G>T | chr16:3731785 | - | 18% |
| *FAT1* | Missense | ENST00000614102:p.Glu2604Lys/c.7810C>T | chr4:186618782 | 33% | 47% |
| *GRIN2A* | Frameshift | ENST00000330684:p.His128fs/c.382_383delCA | chr16:10180028 - 10180029 | 25% | - |
| *GRM3* | Missense | ENST00000361669:p.Asp380Asn/c.1138G>A | chr7:86786930 | 29% | - |
| *HGF* | Missense | ENST00000222390:p.Gly401Arg/c.1201G>C | chr7:81720815 | 25% | - |
| *LRP1B* | Missense | ENST00000389484:p.Lys2376Asn/c.7128G>T | chr2:140598697 | 33% | - |
| *LRP1B* | Splice site | ENST00000389484:c.4170-3delT | chr2:140868265 | - | 33% |
| *NF1* | Missense | ENST00000358273:p.Lys248Arg/c.743A>G | chr17:31182520 | 14% | - |
| *NOTCH1* | Missense | ENST00000277541:p.Gly484Val/c.1451G>T | chr9:136517376 | 50% | - |
| *PCLO* | Missense | ENST00000333891:p.Thr3474Ile/c.10421C>T | chr7:82950167 | 25% | - |
| *PCLO* | Duplication | ENST00000333891:p.Asp2926dup/c.8778_8780insTGA | chr7:82952172 | 83% | 72% |
| *PDE4DIP* | Splice site | ENST00000585156:c.4178-6A>T | chr1:149003608 | 53% | 58% |
| *POLQ* | Missense | ENST00000621776:p.Arg995Gln/c.2984G>A | chr3:121490352 | 22% | - |
| *PRKDC* | Missense | ENST00000314191:p.Glu3682Lys/c.11044G>A | chr8:47785176 | 20% | - |
| *PTPRB* | Missense | ENST00000334414:p.Pro1666Ser/c.4996C>T | chr12:70555307 | - | 29% |
| *PTPRD* | Missense | ENST00000356435:p.Phe921Leu/c.2763C>A | chr9:8486054 | 75% | 27% |
| *RELN* | DelIns | ENST00000424685:GTdelinsTG | chr7:103539147 - 103539147 | - | 65% |
| *RELN* | Missense | ENST00000424685:p.Ala1272Val/c.3815C>T | chr7:103593779 | 33% | - |
| *RELN* | Frameshift | ENST00000424685:p.Ile2769fs/c.8305delA | chr7:103503199 | 25% | - |
| *SLIT2* | Splice site | ENST00000273739:c.1474+2T>G | chr4:20525174 | 100% | 32% |
| *STK11* | Splice site | ENST00000326873:c.375-1C>T | chr19:1219323 | 67% | - |
| *TET1* | Missense | ENST00000373644:p.Gln1638Arg/c.4913A>G | chr10:68681487 | 25% | - |
| *TET1* | Frameshift | ENST00000373644:p.Lys23fs/c.65dupA | chr10:68572395 | 40% | - |
| *ZNF521* | Missense | ENST00000361524:p.Val445Phe/c.1333G>T | chr18:25226585 | 29% | - |

| **Supplementary** **Table 4. Somatic variant profile of Patient 4 tumor tissue** | | | | | |
| --- | --- | --- | --- | --- | --- |
| **Gene** | **Variant Types** | **Variant** | **Chromosomal Location (GRCh38)** | **VAF** | |
|  |  |  |  | **CA** | **SA** |
| *COL2A1* | Missense | ENST00000380518:p.Thr638Ile/c.1913C>T | chr12:47984115 | 31% | 35% |
| *FAT1* | Missense | ENST00000614102:p.Ala4553Gly/c.13658C>G | chr4:186588707 | 34% | 34% |
| *PCLO* | Duplication | ENST00000333891:p.Asp2926dup/c.8778_8780insTGA | chr7:82952172 | 85% | 79% |
| *PDE4DIP* | Splice site | ENST00000585156:c.4178-6A>T | chr1:149003608 | 22% | 27% |
| *SLIT2* | Missense | ENST00000273739:p.Glu1463Ala/c.4388A>C | chr4:20618768 | 48% | 56% |

| **Supplementary** **Table 5. Somatic variant profile of Patient 5 tumor tissue** | | | | | |
| --- | --- | --- | --- | --- | --- |
| **Gene** | **Variant Types** | **Variant** | **Chromosomal Location (GRCh38)** | **VAF** | |
|  |  |  |  | **CA** | **SA** |
| *FAT1* | Missense | ENST00000614102:p.Ser2355Ala/c.7063T>G | chr4:186619529 | 43% | 71% |
| *GRIN2A* | Missense | ENST00000330684:p.Asp344Val/c.1031A>T | chr16:9891077 | 41% | 46% |
| *KMT2D* | Missense | ENST00000301067:p.Ser3708Arg/c.11124C>G | chr12:49033581 | 47% | 35% |
| *NF1* | Missense | ENST00000358273:p.Val54Phe/c.160G>T | chr17:31156082 | 29% | 35% |
| *NF1* | Frameshift | ENST00000358273:p.Leu834fs/c.2500delC | chr17:31229112 | 53% | 50% |
| *PCLO* | Missense | ENST00000333891:p.Ser31Asn/c.92G>A | chr7:83162501 | - | 38% |
| *PCLO* | Duplication | ENST00000333891:p.Asp2926dup/c.8778_8780insTGA | chr7:82952172 | 80% | 83% |
| *PCLO* | Frameshift | ENST00000333891:p.Gln3965fs/c.11892delG | chr7:82916093 | 48% | 42% |
| *PDE4DIP* | Splice site | ENST00000585156:c.4178-6A>T | chr1:149003608 | 54% | 56% |
| *PRKDC* | Missense | ENST00000314191:p.Val1195Ile/c.3583G>A | chr8:47897176 | 38% | - |
| *PTPRD* | Missense | ENST00000356435:p.Val1084Leu/c.3250G>T | chr9:8484282 | 28% | - |
| *TP53* | Nonsense | ENST00000635293:p.Gly227*/c.679G>T | chr17:7673824 | 55% | 75% |

| **Supplementary** **Table 6. Somatic variant profile of Patient 6 tumor tissue** | | | | | |
| --- | --- | --- | --- | --- | --- |
| **Gene** | **Variant Types** | **Variant** | **Chromosomal Location (GRCh38)** | **VAF** | |
|  |  |  |  | **CA** | **SA** |
| *CPS1* | DelIns | ENST00000430249:p.Ile5_Lys6insPhe/c.15_16insTTC | chr2:210556728 | 45% | 34% |
| *CREBBP* | Splice site | ENST00000262367:c.3370-4delT | chr16:3758051 | 46% | - |
| *FAT1* | Missense | ENST00000614102:p.Asp4220Gly/c.12659A>G | chr4:186596887 | - | 73% |
| *FAT4* | Missense | ENST00000394329:p.Ala3896Val/c.11687C>T | chr4:125452703 | - | 51% |
| *GRM3* | Missense | ENST00000361669:p.Tyr196His/c.586T>C | chr7:86786378 | - | 52% |
| *PCLO* | Duplication | ENST00000333891:p.Asp2926dup/c.8778_8780insTGA | chr7:82952172 | 25% | 77% |
| *POLQ* | Splice site | ENST00000621776:c.6179-5dupT | chr3:121483586 | 22% | - |
| *PTPRB* | Missense | ENST00000334414:p.Lys994Glu/c.2980A>G | chr12:70571950 | - | 89% |
| *STK11* | Missense | ENST00000326873:p.Phe354Leu/c.1062C>G | chr19:1223126 | - | 81% |
| *TP53* | Nonsense | ENST00000635293:p.Lys280*/c.838A>T | chr17:7673573 | - | 70% |

**Supplementary** **Table 7. Somatic variant profile in 6 patients**

| **Gene** | **Variant Types** | **Variant** | **Chromosomal Location (GRCh38)** | **Variant Allele Frequency** | | | | | | | | | | | |
| --- | --- | --- | --- | --- | --- | --- | --- | --- | --- | --- | --- | --- | --- | --- | --- |
|  |  |  |  | CA1 | SA1 | CA2 | SA2 | CA3 | SA3 | CA4 | SA4 | CA5 | SA5 | CA6 | SA6 |
| *PCLO* | Missense | ENST00000333891:c.6245C>T(p.Pro2082Leu) | chr7:82954708 | - | - | 40% | - | - | - | - | - | - | - | - | - |
| *PCLO* | Missense | ENST00000333891:c.10421C>T(p.Thr3474Ile) | chr7:82950167 | - | - | - | - | 25% | - | - | - | - | - | - | - |
| *PCLO* | Missense | ENST00000333891:c.92G>A(p.Ser31Asn) | chr7:83162501 | - | - | - | - | - | - | - | - | - | 38% | - | - |
| *PCLO* | Missense | ENST00000333891:c.1405C>A(p.Pro469Thr) | chr7:83155236 | - | 40% | - | - | - | - | - | - | - | - | - | - |
| *PCLO* | Missense | ENST00000333891:c.9053G>A(p.Gly3018Glu) | chr7:82951900 | - | - | - | 25% | - | - | - | - | - | - | - | - |
| *PCLO* | Duplication | ENST00000333891:c.8778_8780insTGA(p.Asp2926dup) | chr7:82952172 | 78% | 71% | 63% | 78% | 83% | 72% | 85% | 79% | 80% | 83% | 25% | 77% |
| *PCLO* | Frameshift | ENST00000333891:c.11892delG(p.Gln3965fs) | chr7:82916093 | - | - | - | - | - | - | - | - | 48% | 42% | - | - |
| *PCLO* | Frameshift | ENST00000333891:c.4181delA(p.Lys1394fs) | chr7:82956771 | - | - | - | 22% | - | - | - | - | - | - | - | - |
| *CPS1* | DelIns | ENST00000430249:ACCDelInsGCT | chr2:210591913 - 210591915 | 45% | 30% | 100% | 100% | - | - | - | - | 51% | 51% | - | - |
| *CPS1* | Missense | ENST00000430249:p.Arg244Gly/c.730C>G | chr2:210590106 | - | - | - | - | 40% | - | - | - | - | - | - | - |
| *CPS1* | DelIns | ENST00000430249:p.Ile5_Lys6insPhe/c.15_16insTTC | chr2:210556728 | 32% | - | 100% | 100% | - | - | - | - | - | - | 45% | 34% |
| *FAT1* | Missense | ENST00000614102:c.8399A>T(p.Gln2800Leu) | chr4:186618193 | - | - | 25% | - | - | - | - | - | - | - | - | - |
| *FAT1* | Missense | ENST00000614102:c.13658C>G(p.Ala4553Gly) | chr4:186588707 | 53% | - | - | - | - | - | 34% | 34% | - | - | - | - |
| *FAT1* | Missense | ENST00000614102:c.7063T>G(p.Ser2355Ala) | chr4:186619529 | - | - | - | - | - | - | - | - | 43% | 71% | - | - |
| *FAT1* | Missense | ENST00000614102:c.11956C>T(p.Gly3986Arg) | chr4:186600051 | - | - | 22% | - | - | - | - | - | - | - | - | - |
| *FAT1* | Missense | ENST00000614102:c.7882G>T(p.Gly2628Cys) | chr4:186618710 | - | 29% | - | - | - | - | - | - | - | - | - | - |
| *FAT1* | Missense | ENST00000614102:c.11962C>T(p.Glu3988Lys) | chr4:186600045 | - | - | 18% | - | - | - | - | - | - | - | - | - |
| *FAT1* | Missense | ENST00000614102:c.7810C>T(p.Glu2604Lys) | chr4:186618782 | - | - | - | 33% | 47% | - | - | - | - | - | - | - |
| *FAT1* | Missense | ENST00000614102:c.12659A>G(p.Asp4220Gly) | chr4:186596887 | - | - | - | - | - | - | - | - | - | - | - | 73% |
| *PDE4DIP* | Splice site | ENST00000585156:c.4178-6A>T | chr1:149003608 | - | - | 33% | - | 53% | 58% | 22% | 27% | 54% | 56% | - | - |
| *PDE4DIP* | Missense | ENST00000585156:p.Glu1752Asp/c.5256A>T | chr1:149009712 | - | 40% | - | - | - | - | - | - | - | - | - | - |
| *PDE4DIP* | Missense | ENST00000585156:p.Arg567Leu/c.1700G>T | chr1:148965578 | - | - | - | 22% | - | - | - | - | - | - | - | - |
| *ARID1B* | Missense | ENST00000636930:p.Glu439Lys/c.1315G>A | chr6:156779244 | - | - | 25% | - | - | - | - | - | - | - | - | - |
| *ARID1B* | Missense | ENST00000636930:p.Met570Ile/c.1710G>A | chr6:156829394 | - | - | - | - | 29% | - | - | - | - | - | - | - |
| *ARID1B* | Missense | ENST00000636930:p.Arg1198Cys/c.3592C>T | chr6:157184357 | - | 33% |  | - | - | - | - | - | - | - | - | - |
| *ARID1B* | DelIns | ENST00000636930:p.Ala460del/c.1379_1381delCGG | chr6:156779296 - 156779299 | - | - | - | - | 25% | - | - | - | - | - | - | - |
| *ARID1B* | DelIns | ENST00000636930:p.Gly328del/c.983_985delGAG | chr6:156778889 - 156778891 | - | - | - | - | - | 13% | 43% | 29% |  |  |  | 19% |
| *CREBBP* | Missense | ENST00000262367:p.Pro2311Ser/c.6931C>T | chr16:3728116 | - | - | 25% | - | - | - | - | - | - | - | - | - |
| *CREBBP* | Missense | ENST00000262367:p.Met2032Ile/c.6096C>T | chr16:3728951 | 25% | - | - | - | - | - | - | - | - | - | - | - |
| *CREBBP* | Missense | ENST00000262367:p.Pro795Ser/c.2383G>A | chr16:3773831 | - | - | - | - | 50% | - | - | - | - | - | - | - |
| *CREBBP* | Missense | ENST00000262367:p.Lys1627Asn/c.4881G>T | chr16:3731785 | - | - | - | - | - | 18% | - | - | - | - | - | - |
| *CREBBP* | Missense | ENST00000262367:p.Gly194Val/c.581G>T | chr16:3850514 | - | 33% | - | - | - | - | - | - | - | - | - | - |
| *CREBBP* | Missense | ENST00000262367:p.Gly1469Arg/c.4405G>A | chr16:3736805 | - | - | - | 29% | - | - | - | - | - | - | - | - |
| *CREBBP* | Splice site | ENST00000262367:c.3370-4delT | chr16:3758051 | - | - | - | - | - | - | - | - | - | - | 46% | - |
| *NF1* | Missense | ENST00000579081:p.Arg31Ser/c.91C>A | chr17:31095298 | - | 33% | - | - | - | - | - | - | - | - | - | - |
| *NF1* | Missense | ENST00000358273:p.Val54Phe/c.160G>T | chr17:31156082 | - | - | - | - | - | - | - | - | 29% | 35% | - | - |
| *NF1* | Missense | ENST00000358273:p.Lys248Arg/c.743A>G | chr17:31182520 | - | - | - | - | 14% | - | - | - | - | - | - | - |
| *NF1* | Missense | ENST00000358273:p.Ile838Ser/c.2513T>G | chr17:31229128 | - | - | - | 33% | - | - | - | - | - | - | - | - |
| *NF1* | Frameshift | ENST00000358273:p.Leu834fs/c.2500delC | chr17:31229112 | - | - | - | - | - | - | - | - | 53% | 50% | - | - |
| *POLQ* | Missense | ENST00000621776:c.2495G>A(p.Cys832Tyr) | chr3:121498540 | - | 40% | - | - | - | - | - | - | - | - | - | - |
| *POLQ* | Missense | ENST00000621776:c.2984G>A(p.Arg995Gln) | chr3:121490352 | - | - | - | - | 22% | - | - | - | - | - | - | - |
| *POLQ* | Missense | ENST00000621776:c.7022C>T(p.Ala2341Val) | chr3:121472091 | - | 25% | - | - | - | - | - | - | - | - | - | - |
| *POLQ* | Splice site | ENST00000621776:c.61795dupT | chr3:121483586 | - | - | 23% | 33% | - | - | - | - | - | - | 22% | - |
| *POLQ* | Splice site | ENST00000621776:c.61796_61795delTT | chr3:121483586_121483587 | - | 50% | - | - | - | - | - | - | - | - | - | - |
| *ARID1A* | Missense | ENST00000324856:c.326C>A(p.Pro109His) | chr1:26696729 | - | - | 33% | - | - | - | - | - | - | - | - | - |
| *ARID1A* | Missense | ENST00000324856:c.4667G>A(p.Gly1556Asp) | chr1:26774894 | 15% | - | - | - | - | - | - | - | - | - | - | - |
| *ARID1A* | Missense | ENST00000324856:c.5679C>G(p.Asp1893Glu) | chr1:26779577 | - | - | - | - | - | 65% | - | - | - | - | - | - |
| *ARID1A* | Missense | ENST00000324856:c.5753G>A(p.Arg1918Gln) | chr1:26779651 | - | 33% | - | - | - | - | - | - | - | - | - | - |
| *ARID1A* | Splice site | ENST00000324856:c.3866+6C>A | chr1:26773502 | - | - | - | 22% | - | - | - | - | - | - | - | - |
| *ARID1A* | DelIns | ENST00000324856:c.567_569delCGG(p.Gly190del) | chr1:26696959_26696961 | - | - | 22% | - | - | - | - | - | - | - | - | - |
| *LRP1B* | Missense | ENST00000442974:p.Pro163Ser/c.487C>T | chr2:140269335 | - | 25% | - | - | - | - | - | - | - | - | - | - |
| *LRP1B* | Missense | ENST00000389484:p.Lys2376Asn/c.7128G>T | chr2:140598697 | - | - | - | - | 33% | - | - | - | - | - | - | - |
| *LRP1B* | Missense | ENST00000389484:p.Cys2951Ser/c.8852G>C | chr2:140495747 | - | - | - | 29% | - | - | - | - | - | - | - | - |
| *LRP1B* | Splice site | ENST00000389484:c.4170-3delT | chr2:140868265 | - | 23% | 36% | - | - | 33% | - | - | - | - | - | - |
| *NOTCH1* | Missense | ENST00000277541:c.2185G>A(p.Ala729Thr) | chr9:136514532 | 29% | - | - | - | - | - | - | - | - | - | - | - |
| *NOTCH1* | Missense | ENST00000277541:c.1451G>T(p.Gly484Val) | chr9:136517376 | - | - | - |  | 50% | - | - | - | - | - | - | - |
| *NOTCH1* | Missense | ENST00000277541:c.889G>A(p.Asp297Asn) | chr9:136518801 | - | - | - | 29% | - | - | - | - | - | - | - | - |
| *NOTCH1* | Missense | ENST00000277541:c.6962A>G(p.Asn2321Ser) | chr9:136496777 | - | 40% | - |  | - | - | - | - | - | - | - | - |
| *NOTCH1* | DelIns | ENST00000277541:c.7244_7246delCAC(p.Pro2415del) | chr9:136496492 | 22% | - | - |  | - | - | - | - | - | - | - | - |
| *NOTCH1* | DelIns | ENST00000277541:c.1189_1201insGGCAGGTGCAGAT(p.Pro401fs) | chr9:136518190 | - | - | - | 33% | - | - | - | - | - | - | - | - |
| *PTPRD* | Missense | ENST00000356435:p.Val1511Met/c.4531G>A | chr9:8376066 | - | 25% | - | - | - | - | - | - | - | - | - | - |
| *PTPRD* | Missense | ENST00000356435:p.Val1084Leu/c.3250G>T | chr9:8484282 | - | - | - | - | - | - | - | - | 28% | - | - | - |
| *PTPRD* | Missense | ENST00000356435:p.Phe921Leu/c.2763C>A | chr9:8486054 | - | - | - | - | 75% | 27% | - | - | - | - | - | - |
| *PTPRD* | Splice site | ENST00000356435:c.551-5C>T | chr9:8526649 | - | 50% | - | 14% | - | - | - | - | - | - | - | - |
| *COL2A1* | Missense | ENST00000380518:p.Lys44Asn/c.132G>T | chr12:48000079 | 22% | - | - | - | - | - | - | - | - | - | - | - |
| *COL2A1* | Missense | ENST00000380518:p.Thr638Ile/c.1913C>T | chr12:47984115 | - | - | - | - | - | - | 31% | 35% | - | - | - | - |
| *COL2A1* | Missense | ENST00000380518:p.Ala1199Thr/c.3595C>T | chr12:47975965 | - | 29% | - | - | - | - | - | - | - | - | - | - |
| *COL2A1* | Splice site | ENST00000380518:c.817-2A>G | chr12:47994049 | - | - | - | - | 33% | - | - | - | - | - | - | - |
| *GRIN2A* | Missense | ENST00000330684:p.His1371Tyr/c.4111G>A | chr16:9763433 | - | - | 14% | - | - | - | - | - | - | - | - | - |
| *GRIN2A* | Missense | ENST00000330684:p.Val1000Met/c.2998C>T | chr16:9764546 | - | 29% | - | - | - | - | - | - | - | - | - | - |
| *GRIN2A* | Missense | ENST00000330684:p.Asp344Val/c.1031A>T | chr16:9891077 | - | - | - | - | - | - | - | - | 41% | 46% | - | - |
| *GRIN2A* | Frameshift | ENST00000330684:p.His128fs/c.382_383delCA | chr16:10180028 - 10180029 | - | - | - | - | 25% | - | - | - | - | - | - | - |
| *KMT2D* | Missense | ENST00000301067:p.Gln2356His/c.7068G>T | chr12:49040702 | - | - | 40% | - | - | - | - | - | - | - | - | - |
| *KMT2D* | Splice site | ENST00000301067:c.5645-4A>G | chr12:49042882 | 33% | - | - | - | - | - | - | - | - | - | - | - |
| *KMT2D* | Missense | ENST00000301067:p.Ser3708Arg/c.11124C>G | chr12:49033581 | - | - | - | - | - | - | - | - | 47% | 35% | - | - |
| *KMT2D* | Missense | ENST00000301067:p.Arg1311Ile/c.3932G>T | chr12:49049193 | - | 20% | - | - | - | - | - | - | - | - | - | - |
| *PTPRB* | Splice site | ENST00000334414:c.980-7C>T | chr12:70596334 | - | - | 33% | - | - | - | - | - | - | - | - | - |
| *PTPRB* | Missense | ENST00000334414:p.Pro1666Ser/c.4996C>T | chr12:70555307 | - | - | - | - | - | 29% | - | - | - | - | - | - |
| *PTPRB* | Missense | ENST00000334414:p.Lys994Glu/c.2980A>G | chr12:70571950 | - | - | - | - | - | - | - | - | - | - | - | 89% |
| *PTPRB* | Nonsense | ENST00000334414:p.Gln2071*/c.6211C>T | chr12:70534645 | - | - | - | 21% | - | - | - | - | - | - | - | - |
| *PTPRB* | Missense | ENST00000334414:p.Arg1933Gln/c.5798G>A | chr12:70538995 | - | 33% | - | - | - | - | - | - | - | - | - | - |
| *SLIT2* | Missense | ENST00000273739:p.Gly1319Asp/c.3956G>A | chr4:20616979 | 25% | - | - | - | - | - | - | - | - | - | - | - |
| *SLIT2* | Missense | ENST00000273739:p.Glu1463Ala/c.4388A>C | chr4:20618768 | - | - | - | - | - | - | 48% | 56% | - | - | - | - |
| *SLIT2* | Splice site | ENST00000273739:c.1474+2T>G | chr4:20525174 | - | - | - | - | 100% | 32% | - | - | - | - | - | - |
| *TP53* | Nonsense | ENST00000635293:p.Gly227*/c.679G>T | chr17:7673824 | - | - | 44% | - | - | - | - | - | 55% | 75% | - | - |
| *TP53* | Nonsense | ENST00000635293:p.Lys280*/c.838A>T | chr17:7673573 | - | - | - | - | - | - | - | - | - | - | - | 70% |
| *TP53* | Splice site | ENST00000413465:c.783-6_783-5delCT | chr17:7662018 - 7662019 | 25% | - | - | - | - | - | - | - | - | - | - | - |
| *FAT4* | Missense | ENST00000394329:p.Asp284Asn/c.850G>A | chr4:125317261 | - | - | 33% | - | - | - | - | - | - | - | - | - |
| *FAT4* | Missense | ENST00000394329:p.Gly3932Arg/c.11794G>A | chr4:125452810 | - | - | - | 25% | - | - | - | - | - | - | - | - |
| *FAT4* | Missense | ENST00000394329:p.Ala3896Val/c.11687C>T | chr4:125452703 | - | - | - | - | - | - | - | - | - | - | - | 51% |
| *FAT4* | Missense | ENST00000394329:p.Ala3822Ser/c.11464G>T | chr4:125452480 | - | 29% | - | - | - | - | - | - | - | - | - | - |
| *GRM3* | Missense | ENST00000454217:p.Pro10Ser/c.28C>T | chr7:86644816 | - | - | 29% | 67% | - | - | - | - | - | - | - | - |
| *GRM3* | Missense | ENST00000361669:p.Tyr196His/c.586T>C | chr7:86786378 | - | - | - | - | - | - | - | - | - | - | - | 52% |
| *GRM3* | Missense | ENST00000361669:p.Asp380Asn/c.1138G>A | chr7:86786930 | - | - | - | - | 29% | - | - | - | - | - | - | - |
| *HGF* | Missense | ENST00000222390:p.Leu19Phe/c.55C>T | chr7:81769917 | - | - | - | 61% | - | - | - | - | - | - | - | - |
| *HGF* | Missense | ENST00000222390:p.Gly401Arg/c.1201G>C | chr7:81720815 | - | - | - | - | 25% | - | - | - | - | - | - | - |
| *HGF* | Missense | ENST00000222390:p.Gly229Val/c.686G>T | chr7:81745060 | - | 29% | - | - | - | - | - | - | - | - | - | - |
| *HGF* | Frameshift | ENST00000222390:p.Thr490fs/c.1468delA | chr7:81710219 | - | 33% | - | - | - | - | - | - | - | - | - | - |
| *PRKDC* | Missense | ENST00000314191:p.Arg3282His/c.9845G>A | chr8:47803383 | 15% | - | - | - | - | - | - | - | - | - | - | - |
| *PRKDC* | Missense | ENST00000314191:p.Val1195Ile/c.3583G>A | chr8:47897176 | - | - | - | - | - | - | - | - | 38% | - | - | - |
| *PRKDC* | Missense | ENST00000314191:p.Glu3682Lys/c.11044G>A | chr8:47785176 | - | - | - | - | 20% | - | - | - | - | - | - | - |
| *PRKDC* | Frameshift | ENST00000314191:p.Asp2376fs/c.7128_7129delCA | chr8:47849379 - 47849380 | - | - | - | 20% | - | - | - | - | - | - | - | - |
| *RELN* | Missense | ENST00000424685:p.Trp652Ser/c.1955G>C | chr7:103650321 | - | 29% | - | - | - | - | - | - | - | - | - | - |
| *RELN* | DelIns | ENST00000424685:GTDelInsTG | chr7:103539147 | - | - | - | - | - | 65% | - | - | - | - | - | - |
| *RELN* | Missense | ENST00000424685:p.Ala1272Val/c.3815C>T | chr7:103593779 | - | - | - | - | 33% | - | - | - | - | - | - | - |
| *RELN* | Frameshift | ENST00000424685:p.Ile2769fs/c.8305delA | chr7:103503199 | - | - | - | - | 25% | - | - | - | - | - | - | - |
| *STK11* | Missense | ENST00000326873:p.Phe354Leu/c.1062C>G | chr19:1223126 | - | - | - | - | - | - | - | - | - | - | - | 81% |
| *STK11* | Missense | ENST00000326873:p.Ala406Gly/c.1217C>G | chr19:1226562 | - | 33% | - | - | - | - | - | - | - | - | - | - |
| *STK11* | Splice site | ENST00000326873:c.375-1C>T | chr19:1219323 | - | - | - | 25% | 67% | - | - | - | - | - | - | - |
| *KEAP1* | Missense | ENST00000437147:p.Glu351Lys/c.1051G>A | chr19:10491617 | 22% | - | - | - | - | - | - | - | - | - | - | - |
| *KEAP1* | Missense | ENST00000171111:p.Val594Met/c.1780G>A | chr19:10486747 | - | - | - | 25% | - | - | - | - | - | - | - | - |
| *KEAP1* | Missense | ENST00000171111:p.Arg204Gln/c.611G>A | chr19:10499423 | - | 29% | - | - | - | - | - | - | - | - | - | - |
| *KRAS* | Missense | ENST00000256078:p.Gly12Cys/c.34G>T | chr12:25245351 | 28% | - | 54% | 37% | - | - | - | - | - | - | - | - |
| *TET1* | Missense | ENST00000373644:p.Gln1638Arg/c.4913A>G | chr10:68681487 | - | - | - | - | 25% | - | - | - | - | - | - | - |
| *TET1* | Frameshift | ENST00000373644:p.Lys23fs/c.65dupA | chr10:68572395 | - | - | - | - | 40% | - | - | - | - | - | - | - |
| *TET1* | Frameshift | ENST00000373644:p.Pro731fs/c.2192delC | chr10:68644918 | - | 33% | - | - | - | - | - | - | - | - | - | - |
| *ZNF521* | Missense | ENST00000361524:p.Val445Phe/c.1333G>T | chr18:25226585 | - | - | - | - | 29% | - | - | - | - | - | - | - |
| *ZNF521* | Missense | ENST00000361524:p.Ser233Phe/c.698C>T | chr18:25227220 | - | 33% | - | - | - | - | - | - | - | - | - | - |
| *ZNF521* | Missense | ENST00000361524:p.Arg602Gly/c.1804A>G | chr18:25226114 | - | - | - | 13% | - | - | - | - | - | - | - | - |
| *APC* | Missense | ENST00000508376:p.Pro2158Ser/c.6472C>T | chr5:112842066 | - | 25% | - | - | - | - | - | - | - | - | - | - |
| *APC* | Missense | ENST00000508376:p.Gly1416Asp/c.4247G>A | chr5:112839841 | - | - | - | - | 33% | - | - | - | - | - | - | - |
| *CDKN2A* | Nonsense | ENST00000498124:p.Trp15*/c.45G>A | chr9:21974783 | - | - | - | - | - | 33% | - | - | - | - | - | - |
| *CDKN2A* | Missense | ENST00000498124:p.Gly122Cys/c.364G>T | chr9:21970995 | - | - | - | - | 40% | - | - | - | - | - | - | - |
| *EPHA5* | Splice site | ENST00000622150:p.Val304Val/c.912C>A | chr4:65495542 | - | 29% | - | - | - | - | - | - | - | - | - | - |
| *EPHA5* | Missense | ENST00000622150:p.Ser451Ile/c.1352C>T | chr4:65490427 | - | 29% | - | - | - | - | - | - | - | - | - | - |
| *FBXW7* | Missense | ENST00000281708:p.Ala204Thr/c.610G>A | chr4:152347046 | 32% | - | - | - | - | - | - | - | - | - | - | - |
| *NFE2L2* | Frameshift | ENST00000397062:p.Asn546fs/c.1637delA | chr2:177230965 | - | 33% | - | - | - | - | - | - | - | - | - | - |
| *PRKCB* | Missense | ENST00000303531:p.Glu591Lys/c.1771G>A | chr16:24191138 | - | 33% | - | - | - | - | - | - | - | - | - | - |

| **Supplementary Table 8. Primary antibodies used in this study** | | | |
| --- | --- | --- | --- |
| **Antibody** | **Clone** | **Dilution** | **Manufacturer** |
|  |  |  |  |
| Mouse Anti-Human CK Monoclonal Antibody | AE1/AE3 | 1 : 500 | Fuzhou Maixin Biotech Co., Ltd. |
| Mouse Anti-Human CK7 Monoclonal Antibody | OV-TL12/30 | 1 : 145 | Fuzhou Maixin Biotech Co., Ltd. |
| Mouse Anti-Human TTF-1 Monoclonal Antibody | SPT24 | 1 : 145 | Fuzhou Maixin Biotech Co., Ltd. |
| Mouse Anti-Human Vimentin Monoclonal Antibody | MX034 | 1 : 150 | Fuzhou Maixin Biotech Co., Ltd. |
| Mouse Anti-Human SMA Monoclonal Antibody | 1A4 | 1 : 100 | Fuzhou Maixin Biotech Co., Ltd. |
| Mouse Anti-Human Desmin Monoclonal Antibody | MX046 | 1 : 150 | Fuzhou Maixin Biotech Co., Ltd. |
| Mouse Anti-Human Myogenin Monoclonal Antibody | F5D | Ready-to-use | Fuzhou Maixin Biotech Co., Ltd. |
| Mouse Anti-Human E-cadherin Monoclonal Antibody | MX020 | 1 : 140 | Fuzhou Maixin Biotech Co., Ltd. |
| Mouse Anti-Human Ki67 Monoclonal Antibody | MXR002 | 1 : 300 | Fuzhou Maixin Biotech Co., Ltd. |
